# *Echinotermes biriba*, a new genus and species of soldierless termite from the Colombian and Peruvian Amazon (Termitidae, Apicotermitinae)

**DOI:** 10.3897/zookeys.748.24253

**Published:** 2018-04-04

**Authors:** Daniel Castro, Rudolf H. Scheffrahn, Tiago F. Carrijo

**Affiliations:** 1 Instituto Amazónico de Investigaciones Científicas SINCHI, Avenida Vásquez Cobo Calles 15 y 16, Leticia, Amazonas, Colombia; 2 Fort Lauderdale Research and Education Center, Institute for Food and Agricultural Sciences, University of Florida, 3205 College Avenue, Davie, Florida 33314, USA; 3 Centro de Ciências Naturais e Humanas, Universidade Federal do ABC, Rua Arcturus 03, Jardim Antares, 09606-070, São Bernardo do Campo, SP, Brazil

**Keywords:** *Anoplotermes*-group, enteric valve, Neotropic, taxonomy

## Abstract

A new Apicotermitinae genus and species *Echinotermes biriba* is described from workers collected on the Andean-Amazon Piedmont in Colombia and Peru. The enteric valve armature of *Echinotermes biriba* Castro & Scheffrahn, **gen. et sp. n.** is a remarkably diagnostic character. A Bayesian phylogenetic analysis using the COI gene and including all other Neotropical Apicotermitinae genera, supports the new genus as a distinct terminal.

## Introduction

The soldierless termites of Amazonia form a dominant group and comprise more than 30% of the termite diversity in neotropical assemblages (Davies 2002, Ackerman et al. 2009, Palin et al. 2011). Although the richness of soldierless taxa is recognized, most have not been described yet (Bourguignon et al. 2015). For example, Palin et al. (2011) list four undescribed *Anoplotermes* species and 18 undescribed species in 13 undescribed genera from Peru. Originally, all neotropical soldierless termites were placed in the genus *Anoplotermes* Müller, 1873. Recognition of much greater taxonomic diversity began with Mathews (1977) who described *Grigiotermes* and *Ruptitermes*, and Fontes (1986) who described *Aparatermes* and *Tetimatermes*. Fontes (1992) provided the first identification key for workers of these five genera. The descriptions of *Longustitermes* (Bourguignon et al. 2010), *Compositermes* (Scheffrahn 2013), *Amplucrutermes*, *Humutermes*, *Hydrecotermes*, *Patawatermes*, and *Rubeotermes* (Bourguignon et al. 2016), and *Disjunctitermes* (Scheffrahn et al. 2017) have advanced the classification of neotropical soldierless taxa but many more remain to be described.

Currently, 13 genera and 52 species of Apicotermitinae are known from the Neotropical region (Bourguignon et al. 2010; Krishna et al. 2013; Scheffrahn 2013; Carrijo et al. 2015; Bourguignon et al. 2016; Scheffrahn et al. 2017). For Colombia, *Anoplotermes ater*, *Anoplotermes parvus*, *Aparatermes silvestrii*, *Humutermes krishnai*, and *Patawatermes turricola* have been reported (Araujo 1977; Constantino 1998; Bourguignon et al. 2016; Pinzón et al. 2017), and Peru records include *Anoplotermes banksi*, *Anoplotermes pacificus*, *Disjunctitermes insularis*, *Rubeotermes jheringi*, and *Ruptitermes reconditus* (Constantino 1998, Bourguignon et al. 2010, Acioli and Constantino 2015, Bourguignon et al. 2016, Scheffrahn et al. 2017). Only 19% of the species of Apicotermitinae of the Neotropics are reported in these two countries.

In this paper *Echinotermes biriba* gen. n. et sp. n. is described based on the morphology of the worker caste and molecular data.

## Materials and methods

The specimens were collected and preserved in 75% or 85% ethanol. The dissection of the enteric valve (EV) was done by removing the P2 tube from the worker’s gut and then expelling all the food particles by means of controlled pressure. The tube was immersed in a PVA medium to completely detach the EV from surrounding muscle tissue and cut longitudinally to splay open the EV for mounting in the medium. The mandibles were also submerged in PVA medium. The terminology used for the worker gut follows Sands (1972) and Noirot (2001).

The COI sequence of *E. biriba* was obtained by DNA extraction and PCR performed by the Canadian Centre for DNA Barcoding following standard high-throughput protocols (deWaard et al. 2008). The PCR employed the primers LepF1 and LepR1 (Hebert et al. 2003) which generated 622 to 652bp of the barcode region of the mitochondrial gene cytochrome c oxidase subunit 1 (COI).

A gene tree was created under Bayesian Inference (BI) using the COI gene. In addition to the sequence of *E. biriba*, a total of 48 GenBank sequences were used: 34 sequences of neotropical Apicotermitinae (21 species, 13 genera), eight non neotropical Apicotermitinae genera, five non-apicotermitine Termitidae, and one Rhinotermitidae, (*Heterotermes crinitus*) as the outgroup. Sequences were aligned under MUSCLE algorithm implemented in Geneious v6.1.6 (Biomatters Ltd., Auckland, New Zealand). Substitution model used (GTR+I+G) was selected through the Akaike Information Criterion (AIC) with the software jModelTest2 (Darriba et al. 2012). The XML input files were generated with BEAUti 1.8.0, and the BI was performed with BEAST 1.8.0 (Drummond et al. 2012). A Yule speciation process, with a random starting tree, and relaxed molecular clock was used as tree priors. Four Markov chain Monte Carlo (MCMC) searches were conducted, each one for 15,000,000 generations, and they were combined to search the most probable tree. Convergence and stationarity were assessed with Tracer 1.5 (Rambaut et al. 2014) and the first 600 trees were discarded as burn-in with TreeAnnotator 1.8.0 and visualized using FigTree 1.3.1.

## Systematics

### Echinotermes

Taxon classificationAnimaliaBlattodeaTermitidae

Castro & Scheffrahn gen. n.

http://zoobank.org/9872DC61-CA8C-42B5-9ABE-62160F532ECD

#### Type-species.

*Echinotermes biriba* sp. n.

**Imago.** Unknown.

#### Description of worker.

**Figure 1.**
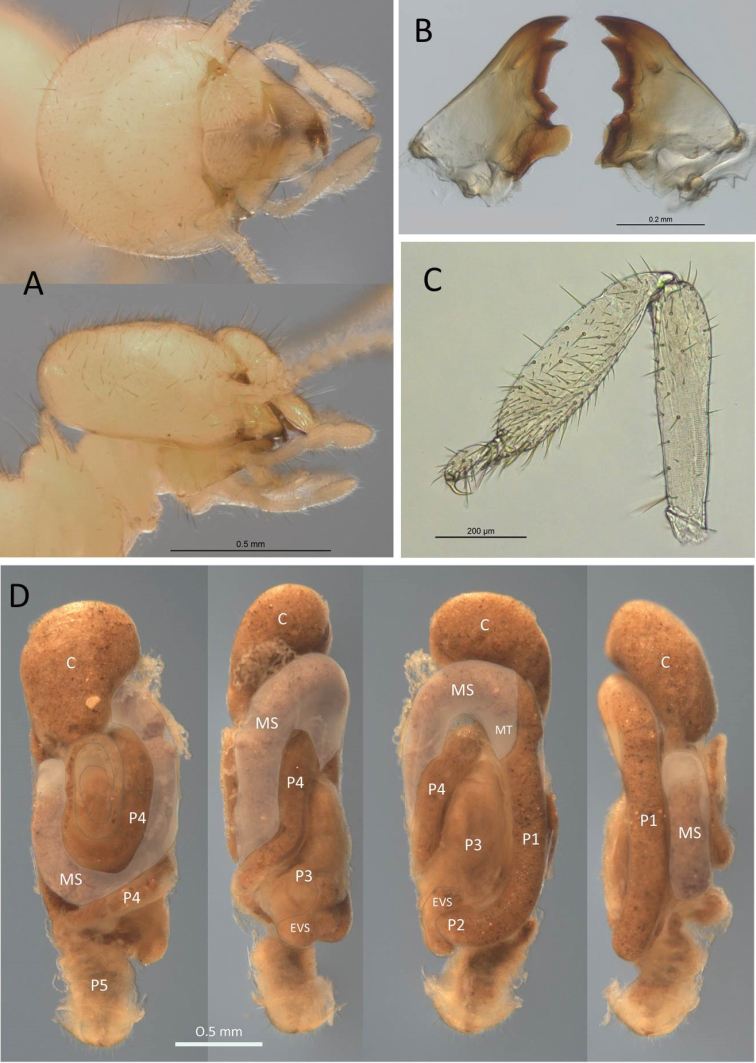
Worker of *Echinotermes biriba* sp. n.: **A** dorsal and lateral views of head and pronotum **B** mandibles **C** right fore-tibia **D** digestive tube from left to right: dorsal, right, ventral and left views. Abbreviations: C = crop, EVS = enteric valve seating, MS = mesenteron, MT = mesenteric tongue, P1–P5 = proctodeal segments.

(Fig. 1). Monomorphic. *Head* capsule and antennae a light yellowish colour; pronotum pale yellow; legs hyaline. Head covered with approx. 30 longer setae (0.1 mm) and approx. 100 shorter setae (≤ 0.05 mm) (Fig. 1A). In lateral view, dorsal surface of the head capsule slightly convex; postclypeus is moderately inflated. Antennae with 14 articles. Pronotum with four or five long setae and numerous short hairs. Mandibles with apical teeth more prominent than first marginal teeth; left mandible with M1+2 equilateral, M3 forming right angle, molar prominence projecting in line with apical tooth; right mandible with concave margin between M1 and M2 (Fig. 1B).

*Fore-tibia* moderately inflated (Fig. 1C) and covered with approx. 60 longer setae and approx. 40 shorter setae; pilosity denser apically. Third (external) spur very small. Femur with approx. 20 sparse large setae. Tibial spurs 2:2:2.

**Figure 2.**
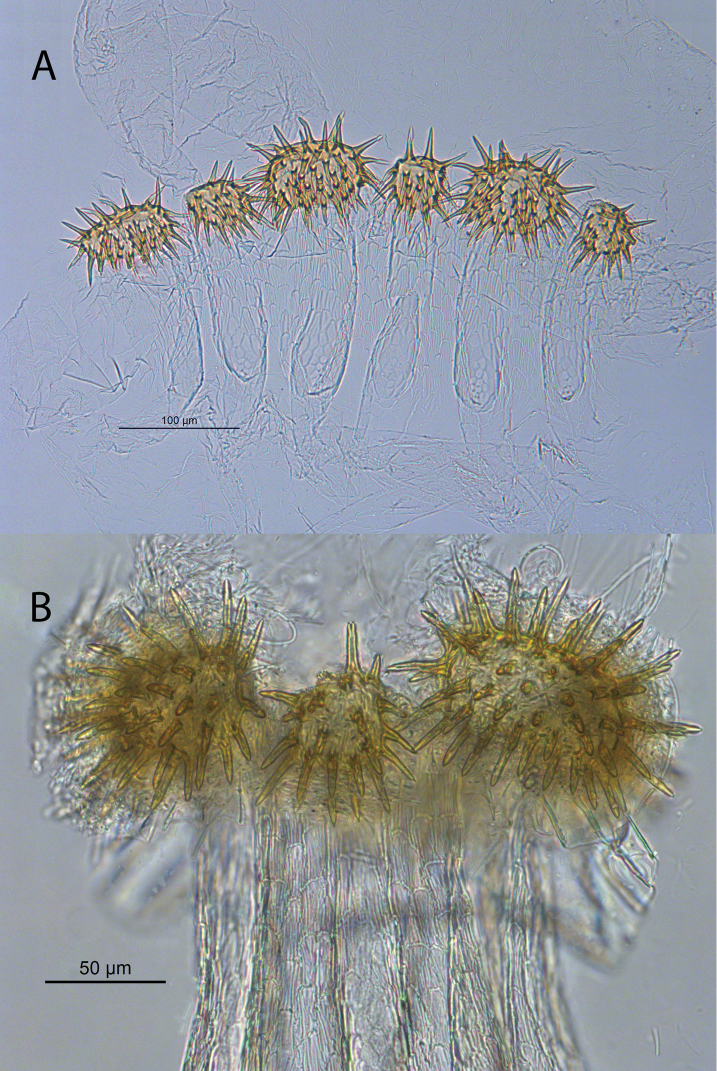
Worker enteric valve of *Echinotermes biriba* sp. n. **A** Spliced mount **B** whole mount, showing *in situ* position of armature. Note the filamentous bacteria attached to the spines. The trilobed seating anterior to the spines (removed in this preparation) is full of bacteria and devoid of food particles, referred by Noirot (2001) as the “bacterial pouch”.

*Digestive tube* (Fig. 1D) with very large crop, more voluminous than paunch (P3). Mesenteron forming complete 360° loop. Mesenteric tongue short, truncate. First proctodeal segment tubular, equal diameter throughout and visible its entire length in ventral view. Enteric valve seating trilobed, with smaller lobe not visible in intact gut. Enteric valve with six cushions, terminating at the opening to the P3 as spiny spheroids (Fig. 2).

#### Diagnosis.

The crop of *E. biriba* is unusually large and the enteric valve armature, consisting of six spherical pectinate pads, is unique among all apicotermitine genera.

#### Remarks.

Mandibles of *Rubeotermes jheringi* and *Humutermes krishnai* are very similar to *E. biriba*, but the first marginal teeth of *E. biriba* are less prominent that those two genera. The diagnostic character of *E. biriba* is the enteric valve armature which is also spiked in the *Humutermes* enteric valve (EV) but in *E. biriba* the EV armature is spherical while in *Humutermes* it is rather flat. *Humutermes* species are smaller than *Echinotermes*. The enteric valve of *Grigiotermes* is composed of six uniform pectinate plates, while in *Patawatermes* the uniform plates are hemispherical.

#### Etymology.

From the Latin *Echino*, meaning spiny, describing the EV armature.

### Echinotermes biriba

Taxon classificationAnimaliaBlattodeaTermitidae

Castro & Scheffrahn sp. n.

http://zoobank.org/9F9BC8F4-57E9-4608-BB48-FBE9E481940B

#### Holotype.

Worker from colony CATAC 2736.

#### Type-locality.

COLOMBIA: Caquetá, Belén de los Andaquíes (1.60794, -75.88683).

#### Paratypes.

PERU: Pasco, Oxapampa, Chatarra forest, (-10.51303, -75.07276), 24/05/2014, 556 m, 14 workers (UF no. PU 144). Additional material: COLOMBIA: Caquetá, Belén de los Andaquíes, Camino Andaquí (1.60794, -75.88683), 31/01/2017, 625 m, 10 workers (CATAC 2736).

#### Description of worker.

(Fig. 1, Table 1) EV armature consists of six prominent spheroids each covered with robust spiny armature; three larger (ca. 30–35 spines) and three smaller (15–20 spines) alternate inside the EV seating. Enteric valve with six unsclerotized cushions some four times longer than wide, each composed of approx. 10–20 ovoid scales.

**Table 1.** Measurements (mm) of ten workers from two colonies of *Echinotermes biriba* sp. n.

	Holotype	PU144		CATAC2736	
		Range	Mean	Range	Mean
Max Head Width	0.74	0.77–0.74	0.75	0.83–0.74	0.78
Pronotum Width	0.44	0.49–0.46	0.48	0.44–0.55	0.51
Hind Tibia Length	0.57	0.53–0.44	0.48	0.61–0.55	0.57
Fore Tibia Length	0.48	0.44–0.35	0.41	0.49–0.43	0.46
Fore Tibia Width	0.13	0.14–0.11	0.12	0.14–0.11	0.12
Fore Tibia Width: Length Ratio	0.27	0.36- 0.24	0.30	0.23–0.28	0.26

#### Diagnosis.

Unique armature of EV composed of alternating larger and smaller spheroids covered with robust spines.

#### Remarks.

See genus remarks above.

#### Ecology and distribution.

In Colombia, *E. biriba* foragers were collected in the same soil sample (0-10 cm depth) with *Longustitermes manni*. Gut contents confirm that *E. biriba* feeds on soil organic matter. This species is only known from the Chatarra forest in the southern Peruvian Amazon, and in a mature secondary forest in the northern Colombian Amazon (Fig. 4).

#### Molecular analysis.

**Figure 3.**
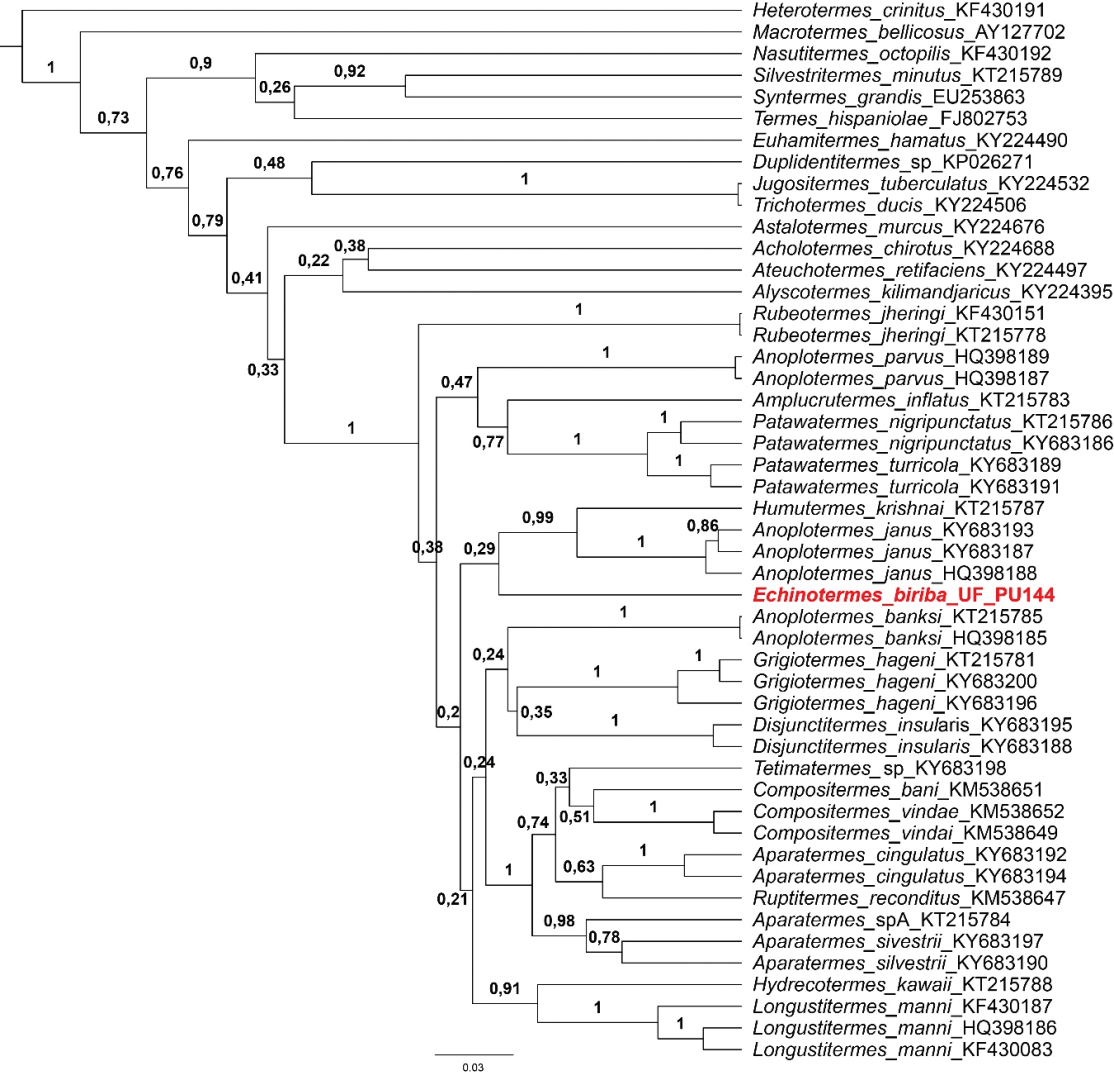
Bayesian gene tree of all described soldierless New World genera using the mitochondrial COI barcode gene showing posterior probabilities. Tree rooted on terminal *Heterotermes crinitus*.

The gene tree recovered the Neotropical Apicotermitinae (NA) as monophyletic, however, the position of *Echinotermes biriba* inside this clade could not be established with this single gene. The low posterior probability of almost every first branching clades in the NA group should be interpreted as a big polytomy, and the new genus as a branch in this polytomy, just as most of the other NA genera (Fig. 3).

**Figure 4.**
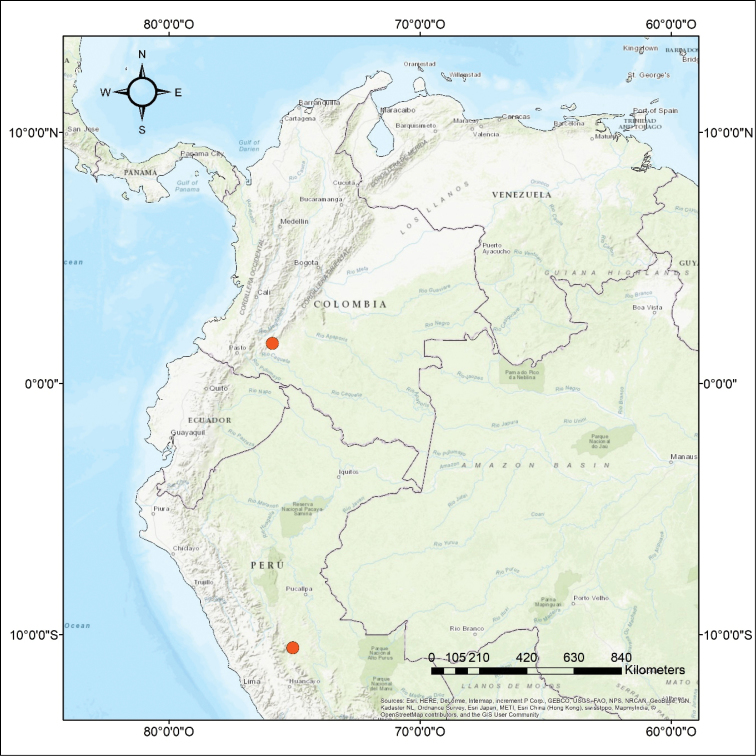
Known localities of *Echinotermes biriba* sp. n.

#### Etymology.

The species name is due to the resemblance of the EV armature with the Amazonian fruit *Rollinia mucosa* (Jacq.) Baill. which is known as “biriba” in the region.

## Discussion

Neotropical soldierless termites have been a taxonomic problem to a large extent because enteric valve (EV) morphology was overlooked. Mathews (1977) showed it was possible to differentiate some New World Apicotermitidae using the EV as had already been done in Africa (Grassé and Noirot 1954, Sands 1972), thus furthering the reclassification of the so-called *Anoplotermes*-group to this day. As with *D. insularis* (Scheffrahn et al. 2017), *E. biriba* is described only from the worker caste with the EV as its most robust diagnostic character.

The Amazon forest contains the greatest diversity of New World termites (Ackerman et al. 2009, Constantino and Cancello 1992), but currently the data show a low diversity of Apicotermitinae compared to other subfamilies such as Nasutitermitinae, Syntermitinae, and Termitinae (Constantino 1991, de Souza and Brown 1994). As new genera and species of neotropical Apicotermitinae are described, the richness of termites, especially in poorly studied countries such as Colombia and Peru will greatly increase.

## Supplementary Material

XML Treatment for Echinotermes

XML Treatment for Echinotermes biriba
