# Corrigendum: *Echinotermes biriba*, a new genus and species of soldierless termite from the Colombian and Peruvian Amazon (Termitidae, Apicotermitinae). ZooKeys 748: 21–30. https://doi.org/10.3897/zookeys.748.24253

**DOI:** 10.3897/zookeys.964.57515

**Published:** 2020-08-27

**Authors:** Daniel Castro, Rudolf H. Scheffrahn, Tiago F. Carrijo

**Affiliations:** 1 Instituto Amazónico de Investigaciones Científicas SINCHI, Avenida Vásquez Cobo Calles 15 y 16, Leticia, Amazonas, Colombia Instituto Amazónico de Investigaciones Científicas Leticia Colombia; 2 Fort Lauderdale Research and Education Center, Institute for Food and Agricultural Sciences, University of Florida, 3205 College Avenue, Davie, Florida 33314, USA University of Florida Davie United States of America; 3 Centro de Ciências Naturais e Humanas, Universidade Federal do ABC, Rua Arcturus 03, Jardim Antares, 09606-070, São Bernardo do Campo, SP, Brazil Universidade Federal do ABC São Bernardo do Campo Brazil

## Abstract

Our recent description of *Echinotermes biriba* (Castro et al. 2018) does not clearly define the type repositories as we only give the acronyms “CATAC” and “UF”. The holotype and paratype workers are deposited in the Colección de artrópodos terrestres de la Amazonía Colombiana of the SINCHI Institute in Leticia, Amazonas, Colombia **(CATAC)**. Additional paratype workers are deposited in the University of Florida Termite Collection at Fort Lauderdale Research and Education Center, Davie, Florida, United States **(UF)**.

Our recent description of *Echinotermes biriba* (Castro et al. 2018) does not clearly define the type repositories as we only give the acronyms “CATAC” and “UF”. The holotype and paratype workers are deposited in the Colección de artrópodos terrestres de la Amazonía Colombiana of the SINCHI Institute in Leticia, Amazonas, Colombia (**CATAC**). Additional paratype workers are deposited in the University of Florida Termite Collection at Fort Lauderdale Research and Education Center, Davie, Florida, United States (**UF**).

## References

[B1] Castro D Scheffrahn RH Carrijo TF (2018) *Echinotermes biriba*, a new genus and species of soldierless termite from the Colombian and Peruvian Amazon (Termitidae, Apicotermitinae). ZooKeys 748: 21–30. 10.3897/zookeys.748.24253 PMC5904502 29674911

